# Innovative Strategies to Overcome Antimicrobial Resistance and Tolerance

**DOI:** 10.3390/microorganisms11010016

**Published:** 2022-12-21

**Authors:** M. Iqbal Choudhary, Ute Römling, Faiza Nadeem, Hafiz Muhammad Bilal, Munirah Zafar, Humera Jahan, Atta ur-Rahman

**Affiliations:** 1 Dr. Panjwani Center for Molecular Medicine and Drug Research, International Center for Chemical and Biological Sciences, University of Karachi, Karachi 75270, Pakistan; 2 H. E. J. Research Institute of Chemistry, International Center for Chemical and Biological Sciences, University of Karachi, Karachi 75270, Pakistan; 3 Department of Microbiology, Tumor and Cell Biology, Karolinska Institutet, 171 65 Stockholm, Sweden

**Keywords:** antibiotics, antimicrobial resistance, antimicrobial tolerance, biofilm, immune response, natural compounds, persister cells

## Abstract

Antimicrobial resistance and tolerance are natural phenomena that arose due to evolutionary adaptation of microorganisms against various xenobiotic agents. These adaptation mechanisms make the current treatment options challenging as it is increasingly difficult to treat a broad range of infections, associated biofilm formation, intracellular and host adapted microbes, as well as persister cells and microbes in protected niches. Therefore, novel strategies are needed to identify the most promising drug targets to overcome the existing hurdles in the treatment of infectious diseases. Furthermore, discovery of novel drug candidates is also much needed, as few novel antimicrobial drugs have been introduced in the last two decades. In this review, we focus on the strategies that may help in the development of innovative small molecules which can interfere with microbial resistance mechanisms. We also highlight the recent advances in optimization of growth media which mimic host conditions and genome scale molecular analyses of microbial response against antimicrobial agents. Furthermore, we discuss the identification of antibiofilm molecules and their mechanisms of action in the light of the distinct physiology and metabolism of biofilm cells. This review thus provides the most recent advances in host mimicking growth media for effective drug discovery and development of antimicrobial and antibiofilm agents.

## 1. Introduction

Antimicrobial agents are produced by almost all organisms, including bacteria, fungi, and humans [1,2,3]. These compounds are synthesized by microorganisms since they exist, not only as protecting agents against other microorganisms, but also as signaling molecules as well as nutrients [4,5,6]. Antimicrobial resistance has only been observed to arise concomitantly locally, excluding mainly resistance against clinically relevant antibiotics in major human pathogens. The massive anthropogenic use of antimicrobial agents in different fields, such as medicine, agriculture, and husbandry including aquafarming has, however, promoted a global spread of resistance against those antimicrobial agents. Emergence of resistance includes the resistance against last resort drugs, such as colistin [7]. Mimicking nature by application of a panel of diverse antimicrobial agents targeting different essential pathways, applied as combinatorial antimicrobial therapy, has been one way to restrict the wider spread of antimicrobial resistance. 

**Figure 1:**
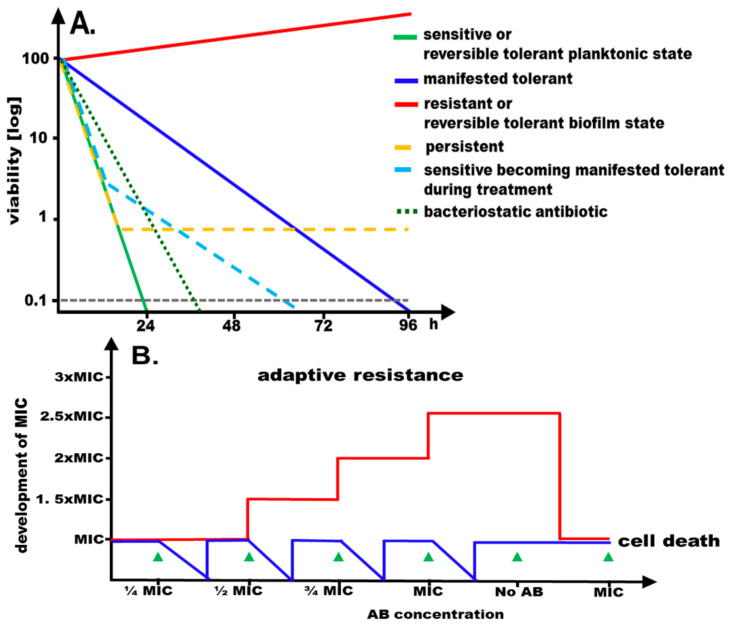
Consequences of exposure to antimicrobials. (**A**) Graphical representation of time-dependent development of viability of a sensitive, resistant, persistent, manifested tolerant and reversible tolerant microbial population to a bactericidal antimicrobial at the minimal inhibitory concentration (MIC). A time course of a hypothetical antibiotic experiments is shown with bacterial cells subjected to the antibiotic at MIC with cellular viability monitored over time. While the majority of cells of a sensitive population are readily killed (>99.9% killing after 24 h; green line), cells of the resistant population continue to grow (red line). Cells of a manifested tolerant population display prolonged viability as compared to sensitive cells (blue line). Upon exposure of a sensitive population with a large fraction of persister cells to the antimicrobial (yellow line), the majority of the cells are killed, but a subpopulation remains viable for an extended period. Upon exposure of a sensitive population to an antimicrobial, manifested tolerance can emerge (light blue line). Biofilms display a reversible tolerance phenotype, showing apparent resistance at the MIC of the planktonic state. The decrease in viability for a bacteriostatic antibiotic is shown for comparison (90–99% killing after 24 h; dotted dark green line). (**B**) Repeated exposure to increasing concentrations of antibiotic below the MIC followed by regrowth can lead to adaptive resistance, which is reversed upon removal of the antibiotic. Red line, development of the minimal inhibitory concentration; blue line, development of viability.

The massive anthropogenic use of antimicrobial agents, detergents, disinfectants, and heavy metals in mono-application contributes significantly to the alteration of the human microflora. Equally has their use led to the emergence of resistance and tolerance phenotypes, which can already arise at subinhibitory concentration of the antimicrobial agent (Figure 1). 

Tolerance is distinct from adaptive and acquired resistance that are defined as enhanced resistance upon exposure to gradually increasing concentrations of the antimicrobials and the acquisition of resistance by mutations and antibiotic resistance genes, respectively. Both modes of resistance significantly alter the minimal inhibitory concentration (MIC) temporarily and permanently, respectively (Figure 1). Reversible tolerance can be displayed by a distinct physiological, yet reversible, state of the organisms such as slow growth or biofilm formation. On the other hand, slowed down killing by the antimicrobial agent at MIC, thereby maintaining significant cell viability, is defined as a manifested mode of tolerance (Figure 1; ref. [8]). A bactericidal effect is only observed at higher concentration of the antimicrobial agent (>32-fold higher as the minimal inhibitory concentration). 

In a wider perspective, the composition and phenotypes of microbial populations in the environment are equally altered, which can be accompanied by the emergence of pandemic clones and enhanced biofilm formation [9,10,11]. Thus, treatment of microbial infections continues to be hampered by major challenges, such as antimicrobial resistance and tolerance. For instance, microbial biofilms, as well as metabolically silent persister cells, a subfraction of cells in a population found predominantly in biofilms, are examples of metabolically altered and downregulated cells, which display extended tolerance (Figure 1; refs. [12,13,14,15]). An immune and antibiotic protected niche for microbes inside host cells and host-matrix embedded biofilms can further lead to recurrent and refractory infections [16,17,18]. Thereby, dissemination of antimicrobial resistance is facilitated by mobile elements, including plasmids, transposons, and integrons. These mobile elements can rapidly thrive within mixed populations consisting of multiple microbial species which are promoted by biofilm formation [10,19]. However, to what extent mutated genomic targets of antibiotic action are horizontally transferred via extracellular matrix DNA (eDNA) or high frequency of recombination status of isolates remains to be determined [20,21]. The formation of biofilms, the build-up of multicellular matrix embedded communities, makes organisms reversibly tolerant towards antimicrobial agents. This tolerance triggers chronic infections which are refractory to diverse antimicrobial treatments. Interestingly, cells forming biofilms are seemingly susceptible to antimicrobials in conventional testing using standard parameters which usually monitors the planktonic state of the organism (Figure 1). Intensive research activities are currently underway to identify novel antimicrobial and antibiofilm agents [22,23,24,25]. These efforts already led to the approval of several novel effective analogs of established and novel classes of antibacterial agents, as well as novel β-lactam/β-lactamase inhibitor combinations, to treat even multi-drug resistant bacterial infections [26,27]. We discuss in this article complementary aspects that might be important for the identification and characterization of novel antimicrobial and antibiofilm agents and their targets. 

## 2. Assessment of Antimicrobial Resistance Mimicking the Host Milieu

Assessment of antimicrobial resistance is conventionally performed by using standardized antimicrobial susceptibility testing [28]. However, these assessment processes are limited as they do not necessarily mimic the antimicrobial susceptibility under host conditions [29]. Comparison of the results of standardized antimicrobial susceptibility testing in vitro with the treatment outcome in patients represent different scenarios with incongruent results, such as in vitro inactive molecules that turn out to be effective in vivo, and vice versa [29,30,31]. For example overcomes a horizontally acquired folate transporter susceptibility of group A streptococci to sulfamethoxazole in vivo due to the acquisition of host folate. This resistance phenotype has been overlooked in conventional susceptibility testing due to the low folate concentration in the growth medium [32]. On the other hand, antibiotics were found effective against multidrug resistant bacteria in animal studies, while no in vitro effect was observed [33]. Furthermore, human biotransformation of clarithromycin produces more potent molecules [34,35], which cannot be predicted by conventional antimicrobial assays. It can thus be concluded that host conditions can alter antimicrobial resistance. Recently, it has been observed that some of these limitations in the assessment of the antimicrobial activity can be overcome by supplementation of the growth medium with bicarbonate which is present in host blood and tissues. Bicarbonate is growth inhibitory towards microorganisms when applied as an individual component at high concentrations. Furthermore, bicarbonate effectively enhanced the susceptibility towards aminoglycoside and macrolide antibiotics at subinhibitory concentrations [36,37,38,39]. Interestingly, enhanced susceptibility was observed even in the presence of an antimicrobial resistance cassette. In the same line, growth of *Escherichia coli,* resistant to the beta-lactam mecillinam, in urine medium partially or fully reverted the antimicrobial resistance of a clinically relevant mutation in the *cysB* gene [40]. Moreover, testing a range of antibiotics under standardized conditions by using Mueller–Hinton Broth versus host-like conditions demonstrated that susceptibility can be substantially different. For example, tissue-dependent high level of antimicrobial resistance in the gastrointestinal pathogen and model organism for typhoid fever *Salmonella typhimurium* was reflected only by the host-like medium [41].

## 3. Assessment of Antimicrobial Tolerance Mimicking the Host Milieu

Another limitation of standardized antimicrobial susceptibility testing arises as usually planktonic cell cultures are assessed for susceptibility. This conventional evaluation method identifies antimicrobial susceptible isolates even if a treatment refractory biofilm infection persists. However, the observed resistance towards treatment is based on tolerance of a microbial biofilm which develops specifically under host conditions in vivo [42]. In order to more closely mimic the relevant host environment, including the altered physiological and metabolic state of microorganisms, more complex semisynthetic (also named as synthetic, artificial, or simulated) growth media were developed (Table 1). Such growth media enable us to assess biofilm formation and to understand the effect of individual medium components on microbial biofilm behavior, including antimicrobial tolerance. The impact and interactions among individual members of a microbial consortium can be investigated in an in vitro set up using a host-simulating growth medium. 

Cystic fibrosis is a genetically inherited disease which is characterized by enhanced lung sputum production with altered composition prone to be colonized by microbes [58]. Persistent *P. aeruginosa* lung infection is one of the key factors which determines morbidity and mortality in cystic fibrosis patients. Enhanced biofilm formation of the microorganisms, due to overproduction of the exopolysaccharide alginate, is the key determinant of persistent infection. Thus, this phenotypic and/or genotypic development represents another example of developed tolerance alternatively resistance towards antimicrobial treatment. Growth of *P. aeruginosa* in artificial sputum medium resembling the cystic fibrosis lung environment (Table 1), including the sputum components, such as mucin and host DNA, leads to a rapid microbial adaptation. These metabolic and physiological adaptations include emergence of small colony variants and enhanced biofilm formation, and thus the microbe displaying enhanced tolerance against antimicrobial agents [45,46,59,60,61]. Interestingly, the biofilm displays mainly as tight cellular aggregates in the artificial sputum medium with few cells attached to the abiotic surface. A similar situation is observed in the cystic fibrosis lung [62], where the microbes reside in the sputum, but do not adhere to the epithelial lining. Sputum components, including amino acids which are present in high concentrations under deteriorating health conditions of the patient, contribute to an enhanced biofilm formation. Thus, a vicious cycle between disease severity and the difficulty to eradicate the microorganisms can be emulated in vitro, as it has been observed in vivo [63]. Consequently, with the toolbox to modulate individual components of an artificial medium in hand, different disease stages and conditions in individual patients, sputum composition reflecting disease severity, as well as environmental conditions, including the contribution of the accompanying microflora, can be mimicked [61,64,65]. Furthermore, the artificial sputum medium can help in understanding the genetic components of the bacterial isolates required for growth [66,67]. Therefore, use of host-like conditions in microbial susceptibility testing, including biofilm-related tolerance, supports in identifying specific drug targets, as well as unraveling the bacterial physiology, along with physiological and metabolic basis of biofilm formation [61,68]. By using this approach, growth, biofilm formation, and antimicrobial tolerance have not only been assessed for *P. aeruginosa*, but also for other microorganisms colonizing the cystic fibrosis lung, including *Stenotrophomonas maltophilia*, *Mycobacterium abscessus,* and black yeast [69,70,71]. It is worth mentioning that the antimetabolite oxythiamine was initially identified as an antimicrobial agent in artificial sputum medium, showing synergistic effects in combination with tetracycline for which *P. aeruginosa* is intrinsically resistant [72]. It remains to be shown whether the recent pharmacological restoration of functionality of the cystic fibrosis transmembrane conductance regulator CFTR, that leads to diminished growth of pathogens and re-establishment of a conventional microflora, can be mimicked by altering the composition of the artificial sputum medium. Artificial medium use reflecting distinctly similar host conditions can also be applied to specifically monitor bacterial colonization in primary ciliary dyskinesia and chronic obstructive pulmonary diseases (COPD) [73,74]. 

Biofilms formed under conditions simulating the host environment are not only morphologically different, but also follow different genetic programs [75]. Artificial urine medium, simulated synovial fluid, chronic wound medium, and simulated colonic environment medium form a panel of growth media developed to mimic specific infectious disease situations more closely (Table 1; refs. [48,54,76]). For example, in chronic wound medium, biofilms exhibit enhanced resistance to disinfectants and integrated anaerobes have been recovered from chronic wounds [53,54]. In such a medium, the physiology of microorganisms is significantly altered and relevant wound microbes, such as *P. aeruginosa* and *S. aureus*, have been found to be less virulent [77]. These in vitro observations are consistent with the fact that sepsis does not develop from chronic wound infections. Growth in the chronic wound medium also demonstrated a beneficial effect of co-infection of *S. aureus* and *P. aeruginosa,* including enhanced antimicrobial tolerance. Furthermore, distinct disease situations such as urinary tract and cystic fibrosis lung infection, provide particular growth conditions for the pathogen. Consequently, upon growth in different artificial media, perhaps not surprisingly, specific mutations can be selected upon exposure to antimicrobials. Upon selection, mutations leading to resistance towards fosfomycin, an epoxid-based cell wall inhibiting antibiotic, could be distinct in *S. maltophilia* grown either in urine or synthetic sputum medium [78]. Undoubtedly, the growth of microbial isolates in urine, sputum, saliva, or synovial fluid, derived directly from patients or animals, is an excellent approach [79,80,81]. The use of synthetic media has, however, the advantage that the effect of individual components in the medium on biofilm formation and antimicrobial tolerance can be assessed and correlated with different patients’ conditions. Besides the alteration of the growth medium, the properties of the biotic and abiotic surfaces can be determinants of the type of biofilm formed. For example, initial bacterial adhesion as a first step to develop dental plaque biofilm on a biological apatite surface is not affected by electrostatic repulsive forces [82]. Biofilm formation in osteomyelitis has been mimicked using bone blocks from bovine femur [83]. Multiple microbes adhere to central venous and urinary catheters made of silicone to develop a biofilm that eventually contains pathogens [84]. Dentures made of acrylic resins select for *Candida albicans* biofilm formation [85]. Surface roughness was identified as one parameter which is a determinant of initiation of biofilm formation [86]. Furthermore, static and dynamic (continuous flow with constant renewal of the medium) biofilm models have also been developed to reflect differential access to nutrients and the removal of waste products in different systems [87].

## 4. Innovative Strategies to Overcome Antimicrobial Resistance

Many of the established antimicrobials target core functions essential for viability, such as DNA (quinolones), RNA, and protein (aminoglycosides) synthesis, cell wall biosynthesis (β-lactams, fosfomycin and vancomycin), and outer membrane integrity, whereas a few are targeting central metabolism. In order to overcome antimicrobial resistance, effective antibiotics are isolated from natural resources [88,89]. This approach is complemented by the discovery of novel antimicrobial agents based on metabolic or virulence targets, host-adapted screening approaches, biotransformation, as well as machine learning approaches, along with genetic information of biosynthesis modules that can be edited to synthesize novel compound [90,91,92,93,94,95,96]. Additional strategies include the combinatorial screening of existing drugs in order to enhance the efficiency of antimicrobial agents through synergistic effects [97]. The discovery of intrinsic antimicrobial resistance components [98,99] might help in the identification of novel drug targets [100,101]. Such sensitizes specifically the loss of the muramyl endopeptidase Spr the Gram-negative bacterium Salmonella typhimurium against vancomycin [102]. Alternatively, upon already underlying multidrug resistance, the identification of novel therapeutic targets can be achieved, for example, by identification of components required for virulence or persistence. Candidate compounds can then be identified by molecular docking, virtual prediction of small molecule binding sites, to the protein’s crystal structure or to its structural model.- More unbiased approaches include binding studies using cell extracts or a compound library screening in vitro, following expression and purification of validated protein targets [103]. Alternatively, already addressed targets can be employed to develop new drugs, through approaches such as modulation of existing antimicrobial agents in case of altered or homologous targets [94]. This is an especially useful approach as multidrug resistant microbes can persist in the environment and do not readily reverse even upon discontinuation of the treatment [104]. For example, N-thiol substituted monocyclic β-lactams covalently inhibits abundant L-D transpeptidase 2 which performs 3,3-diaminopimelic acid crosslinks in peptidoglycan of *Mycobacterium tuberculosis* thereby being effective against dormant and multidrug resistant isolates [105]. It has also been observed that the frequency of horizontal plasmid transfer increases in the presence of subinhibitory concentrations of antibiotics [106]. Thus, targeting of plasmid maintenance or their conjugation can be novel strategies to lower the frequency of antimicrobial resistance, as exemplified with an IncFIA plasmid [107]. Such strategies, if sufficiently specific for multidrug resistance plasmids, might also be appropriate to remove resistance plasmids or to prevent their transfer from and between the commensal flora.

## 5. Innovative Strategies to Overcome Antimicrobial Tolerance in Biofilms

Biofilm formation is a major cause of tolerance against antimicrobial treatment [12,14]. Thereby, multiple characteristics of biofilms have been identified which contribute to antimicrobial tolerance. For instance, production of the extracellular matrix serves as physical and chemical barriers [108], induction of reactive oxygen species by antibiotic treatment is less efficient [109], and the metabolism of biofilms is significantly altered and slowed down in chronic infections [110], including the presence of a substantial fraction of metabolically silent persister cells [111]. As such, depletion of the extracellular biofilm matrix by nucleases and/or proteases can enhance the efficiency of antimicrobial treatment [108]. The degradation of the extracellular biofilm matrix by hydrolyzing enzymes is highly efficient. Indeed, production of extracellular matrix components can positively affect *P. aeruginosa* biofilm formation, and the synthesis of the second messenger cyclic di-GMP, a ubiquitous activator of biofilm formation [112]. Thus, the degradation of the biofilm matrix is not only a simple removal of physical and chemical barriers, but also involves the downregulation of biofilm physiology and metabolism, and hence contributes to the success of removal of the extracellular biofilm matrix as a therapeutic strategy.

A few clinically relevant antimicrobial agents, rifampin and fluoroquinolones, have shown to be effective against Gram-positive and Gram-negative biofilm infections, respectively [113]. Antibiofilm agents, including compounds that even disperse already established biofilms, are widespread in nature and have been developed into effective antibiofilm agents (Table 2; refs. [114,115,116]). For example, sensing of the innate immune agent nitric oxide (NO) at subinhibitory concentration disperses biofilms in a broad range of bacterial species, including human pathogens (Table 2; refs. [117,118]). Biofilm dispersal seems to be affected through downregulation of the second messenger cyclic di-GMP by distinct protein members of conceptually similar signaling pathways. In representative isolates belonging to different species, NO binds to an *N*-terminal or free-standing signaling domain that subsequently activates a cyclic di-GMP specific phosphodiesterase. With several clinical trials under way (NCT02498535; refs. [119,120]), success in the application of NO as an antibiofilm agent might be based on a combination of antimicrobial, antibiofilm, and host physiological effects [121]. The antimicrobial peptide LL-37 has been shown to possess a potent antibiofilm, rather than antimicrobial activity against various pathogens, such as *P. aeruginosa* and *Escherichia coli* (Table 2; refs. [122,123]). A recently developed antimicrobial peptide–vancomycin conjugate combined the antibiofilm with antimicrobial and immunostimulatory effects to reduce bacterial load in an in vivo abscess model [124]. The human hormones brain natriuretic peptide (hBNP) and C-type natriuretic peptide (hCNP) equally efficiently inhibit biofilm formation of *P. aeruginosa* at concentrations over 1000-fold lower than their antimicrobial concentration (Table 2). On the other hand, a highly differential temperature-dependent effect on biofilm formation of Gram-positive pathogens has been observed with human atrial natriuretic peptide (hANP) and hCNP to inhibit biofilm formation of *S. aureus* at body temperature. Various established antimicrobial agents, such as macrolides, have been reported to affect biofilm formation at subinhibitory concentrations, although not necessarily through the same mechanism or extent in different microbial species (Table 2; ref. [125]). While macrolides selectively affect the translation of messenger RNA into proteins by interacting with the 23S RNA of ribosomes, and prevent 50S ribosomal subunit assembly [126], transcriptional profiling of the response against subinhibitory concentrations of the semi-synthetic macrolide clarithromycin to prevent ancient rdar biofilm formation of *Salmonella typhimurium*, a biofilm directed by the transcriptional regulator CsgD via the expression of amyloid curli fimbriae and the exopolysaccharide cellulose, indicated selective upregulation of ribosomal subunit genes with their gene products potentially interacting with clarithromycin. Upregulation of the heat shock stress response with folding and holding chaperons is also indicative of impaired protein homeostasis [127]. Transcriptional analysis further indicated the redirection of microbial metabolism towards an oxygen- and energy-depleted status where energy is derived from L-arginine catabolism and propane-1,2-diol and ethanolamine degradation rather than by oxidative phosphorylation. This defined response might not only be explained by the macrolide clarithromycin to differentially inhibit ribosome assembly or translation, but also indicate an off-target activity of the macrolide antimicrobial agent.

The second messenger cyclic di-GMP signaling network is a major determinant of tolerance against antimicrobials and detergents [152,153]. Alternative strategies to combat biofilm formation by mimicking natural situations include direct targeting of the cyclic di-GMP signaling molecules by complexing peptides with high affinity binding sites of protein receptors [154]. Although small molecule compounds can interfere with a variety of regulatory or biosynthetic biofilm pathways, screening identified a hydrazonodiaminopyrazole derivative, (Z)-4-(2-(3-fluorophenyl)hydrazineylidene)-5-imino-4,5-dihydro-1H-pyrazol-3-amine, that activates the breakdown of the cyclic di-GMP biofilm activator by activation of a cyclic di-GMP specific phosphodiesterase, leading to biofilm dispersal [150]. Furthermore, high spider-like biofilms, formed by clinical isolates of the yeast *Candida parapsilosis,* can be selectively targeted with a benzophenone semicarbazone derivative to elicit a defined transcriptional response (Nadeem, Shafeeq et al., manuscript in preparation). Similar analyses explaining the antibiofilm effect on a molecular level will help in the identification of novel antimicrobial and antibiofilm targets. These strategies combined with transcriptional profiles of alternative antibiofilm and antimicrobial agents can lead to the development of rationalized combinatorial strategies against biofilm infections [155].

An additional challenge during chronic infections is the presence of so-called small colony variants, comprised of mutated cells with metabolic downregulation, and enhanced antimicrobial resistance, and biofilm formation [156,157,158,159]. Mutations that lead to the emergence of small colony variants can occur in the heme and menaquinone biosynthesis pathways, which leads to impairment of functionality of the respiratory chain, in carbonic anhydrases (that fix inorganic CO_2_), and in the de novo pyrimidine biosynthesis pathway [160]. Targeting small colony variants might require novel experimental approaches as their metabolism and physiology, including elevated biofilm formation, is substantially different. Metabolic downregulation leading to antimicrobial tolerance is also displayed by some slow growing microorganisms. Such organisms can be resensitized by metabolic stimulation through nutrients [129]. Induction of dormancy can also be prevented by pharmacological interference [129] to stimulate metabolism and respiration and to increase the proton motif force required for the uptake of some classes of antimicrobial agents.

## 6. Holistic Assessment of Antimicrobial Tolerance

**Figure 2:**
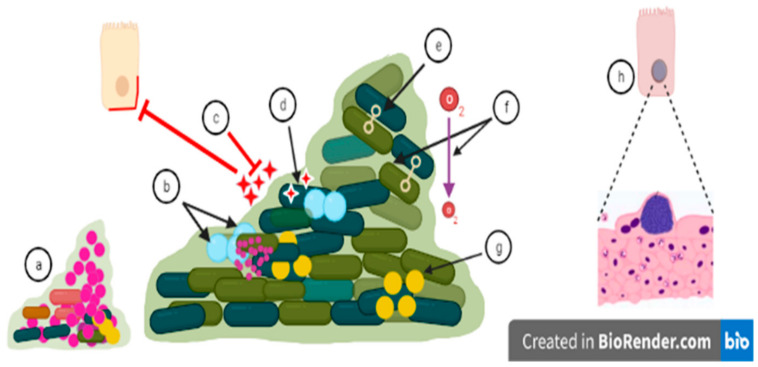
Biofilm related treatment challenges: (a) Small colony variants are mutants arising during chronic infections due to, for example, a defect in the heme biosynthesis pathway. Small colony variants possess increased antibiotic resistance. (b) Growth rate is not uniform inside biofilm as persister cells are non-growing cells, and antibiotics may be inactive against these cells with low metabolic activity. (c) Antimicrobials fail to penetrate into the surface layer of biofilms or might be inactivated by extracellular matrix components, which decreases the effectiveness of antimicrobials. An increasing dose of the antimicrobial may lead to a cytotoxic effect on host cells [165]. (d) Antibiotics can be degraded or destroyed by enzymes which are secreted into the biofilm matrix. (e) Biofilm formation increases the effectiveness of genes transfer among the microbes constituting the biofilm [166]. Biofilm formation thus facilitates the transfer of antibiotic-resistant genes from resistant microbial strains to susceptible microbial strains. (f) pH and oxygen levels are different in different microenvironments of the biofilm which aid the defensive mechanisms of biofilm and can prevent uptake of antimicrobial agents. (g) Microbial cells communicate through quorum sensing which can trigger increased virulence and biofilm formation by altered gene expression, and accelerating the process of antibiotic tolerance [167]. This mechanism can stimulate biofilm formation, but also disperse biofilms depending on the autoinducer molecule. (h) Intracellular biofilm as, for example, observed in the murine cystitis model. After microbial invasion, these biofilm-like intracellular bacterial communities replicate and persist in host cells, protected from antimicrobial action and can subsequently disperse to other host cells [168].

Although effective against microbial cells, several conventionally used antibiotics, such as beta-lactam antibiotics or the aminoglycoside gentamicin, do not penetrate into host cells, and thus fail to reach to intracellular bacteria (Figure 2; ref. [161]). A long-overlooked niche leading to recurrent infections is the intracellular presence of microbes which are evolutionarily not considered to possess an intracellular lifestyle. For example, uropathogenic *E. coli* survives antimicrobial treatment as an intracellular biofilm in bladder epithelial cells [16], while *Staphylococcus aureus* invades and survives in phagocytic and non-phagocytic host cells [162]. Antimicrobial agents, such as the fluoroquinolone enrofloxacin, might be poorly effective against intracellular microbes due to insufficient intracellular accumulation. They may also be less effective against microbes which reside in immunologically favorable niches in the host tissues [163,164]. Thus, besides being an effective antimicrobial agent against free-living planktonic and/or biofilm-forming microbes and non-cytotoxic against host cells, accumulation of antimicrobial agents in host cells, their tissue penetration, and immunomodulatory characteristics might add to the success of antimicrobial therapies. 

Antimicrobial agents often have additional biological effects, besides their bactericidal or bacteriostatic effect on the targeted microbes [169]. Such antibiotics can show cross-effectiveness against highly homologous structures present in alternative microbes, fungi, parasites, and helminths and can also exert a substantial toxicity against host cells. This prevents their systemic use for the treatment of infections in humans [170,171,172,173]. Additional adverse effects, such as antibiotic allergy [174] or the selection for alternative pathogens [175], might be experienced upon long-term use. For example, *M. abscessus* emerged as an opportunistic pathogen in chronic lung infection in immunocompromised individuals [176]. 

The aminoglycoside antibiotic, nourseothricin, a natural product of the streptothricin class, is composed of a streptolidine, carbamyolated gulosamine, and variable number of β-lysine moieties. This antibiotic causes miscoding by a potentially unique binding mechanism to the ribosome [177,178]. Nourseothricin is effective not only against bacteria, but also against other microorganisms, such as archaea, fungi, protozoa, and microalgae. However, its toxicity against eukaryotic cells prevents its clinical use [179,180,181]. The effect of antimicrobial agents not only on pathogens but on the commensal microflora might increase the risk of viral [182,183] and fungal infections [184]. Besides cytotoxic and cytostatic effects beyond the primarily targeted microbes, antimicrobial agents can have a substantial modulating effect on host metabolism and physiology. Furthermore, antibiotics can affect immune responses, which might substantially alter the outcome of the antimicrobial treatment. For example, some antimicrobial agents, such as the macrolide clarithromycin, accumulate in host cells and provoke an immunomodulatory effect which contributes towards improved conditions [185]. Intriguingly, it has recently been shown that treatment success can be enhanced by stimulation of the immune system, especially in immune compromised host niches [163]. Similar strategies, in combination with strong antibiofilm agents, might also enhance the success of antimicrobial therapies against biofilm infections [107]. 

## 7. Discussion and Conclusions

The discovery of antimicrobial agents and their extensive use in the treatment of acute infections has been a major milestone in medical sciences. However, their broad spectrum uses from therapeutics to agriculture and including poultry, cosmetics, and aquaculture have led to the emergence of multidrug resistance in targeted pathogens. As a result, this major therapeutic achievement might be lost with consequences beyond the eradication of acute infections. Not only does immediate effective antimicrobial therapy target acute infections, but also follow-up diseases such as autoimmune diseases, e.g., rheumatic heart disease, can be alleviated or even prevented. Subclinical microbial infections also cause or are associated with lifestyle diseases, such as atherosclerotic cardiovascular disease due to enhanced gut permeability and poor dental health. There are indications that antimicrobial treatment leads to improvement in such situations. Similarly, treatment of chronic infections, often associated with biofilm formation as a major virulence factor, is increasingly challenging as the raise in medical care that requires indwelling devices becomes more frequent. Equally difficult is the treatment of infections in immunocompromised individuals including individuals with diabetes and cancer patients undergoing chemotherapy who are prone to a diversity of microbial infections, more frequently and with more severe outcomes. Antimicrobial agents to treat immunocompromised individuals ideally are bactericidal to kill microbes fast and effectively as the immune system cannot aid in the eradication of the organisms. Tailor-suited immunostimulatory therapies might be able to support eradication and prevent reinfection. An additional challenge is the evolution of tolerant microorganisms manifested by specific mutations to subsequently promote the development of resistance and recurrent infections [8,186] equally as the emergence of reversibly tolerant microorganisms that display enhanced biofilm formation [11]. Thereby, manifested tolerance resembles the phenotype observed with a bacteriostatic antibiotic. Whether either of these two modes of tolerance is increasing among the various pathogens requires further investigation and clinical studies. Thus, multiple strategies need to be deployed in order to develop novel and effective treatment approaches. Antibiofilm approaches might, however, be challenging to develop due to the complexity of biofilm regulation in combination with tolerance. As such, small molecular compounds might have a differential and even opposite effect on biofilm formation of different species of the human microbiome. Among the most promising approaches, molecular genome wide approaches help to identify novel targets of intrinsic antimicrobial resistance. This can aid in identifying species or even strain-specific antimicrobial targets and antibiofilm approaches which can target ubiquitous second messenger pathways mediating biofilm formation, small colony variants, and/or persister cell formation. Genome mining of human and microbial resources, on the other hand, has already provided novel opportunities to discover novel classes of antimicrobial and antibiofilm agents [187,188]. 

## Figures and Tables

**Table 1:** Growth media closely simulating host conditions.

Host Condition Simulating Medium	Simulated Host Condition	Infection Condition Investigated	Reference
Ex vivo pig lung (EVPL)	Cystic fibrosis lung	Chronic cystic fibrosis biofilm infections, *Klebsiella pneumoniae*-triggered pneumonia	[43]
Artificial Sputum Medium (ASM)	Cystic fibrosis sputum	Lung infection, microcolony formation, antimicrobial resistance, adaptation of *Pseudomonas aeruginosa* and other relevant microbes to host conditions	[44]
Synthetic cystic fibrosis sputum medium (SCFM)	Cystic fibrosis sputum	Microbial physiology, biofilm formation, and antimicrobial resistance of *P. aeruginosa* and *K. pneumoniae*-triggered pneumonia	[45,46]
Porcine vaginal mucosa (PVM)	Human vaginal mucosa	*Staphylococcus aureus* biofilm formation	[47]
Artificial Urine Medium (AUM)	Urine	Urinary tract infection	[48]
Simulated Ileal Environment Media (SIEM)	Ileac environment	Gut infections—small intestine	[49]
Simulated Colonic Environment Media (SCEM)	Colonic environment	Gut infections—large intestine	[49]
Cholesterol coated surfaces	Gall stones	Colonization of gall stones	[50]
Gallstones in bile	Gall stones	Colonization of gall stones	[51]
Chronic wound mimicking medium	Interstitial fluid	Tissue infections, chronic wound infections	[52,53,54]
Synthetic synovial fluid	Synovial fluid	Biofilm formation of *S. aureus* mimicking infection of prosthetic joints	[55]
Intracellular medium	*Salmonella* containing vacuole	Intracellular growth	[56]
Mucin supplemented medium	Intestinal environment	Growth in small and large intestine	[57]

**Table 2:** Selected identified anti-biofilm agents and their major physiological effects on the targeted microbes and the host.

S. No	Antibiofilm Agent	Source of Compound	Active against	Effect on Biofilm/Mechanism	Alternative Physiological Activities
**Natural compounds**					
**1.**	Ellagic acid glycosides	Plant (*Rubus ulmifolius* Schott; *Euphorbia humifusa, Punica granatum*, *Fragaria)*	*S. aureus*	Inhibits cellular adhesion [128]	-
**2.**	Carolacton	Microbe (*Sorangium cellulosum*)	*Streptococcus mutans*	Decreases cell viability in biofilm [128]	Inhibits human cancer cell lines MTHFD 1 and MTHFD 2 [128]
**3.**	Promysalin	Microbe (*Pseudomonas putida*)	*P. aeruginosa*	Growth inhibitory against MDR *Pseudomonas* spp.; affects succinate dehydrogenase [128]	Can enhance biofilms; inhibits pyoverdine production in *Pseudomonas* spp. [128]
**4.**	Flustramine C and its derivatives	Marine colonial animal (*Flustra foliacea*)	*Acinetobacter baumannii*, *E. coli*, and MRSA *S. aureus*	Inhibition of biofilm formation, potentially through indole pathways [128]	-
**5.**	Meridianin D and derivatives	Marine tunicate (*Aplidium meridianum*)	MRSA *S. aureus*	Inhibits and disperses biofilms [128]	Inhibits some kinases [128]; Synergistic with colicin in colicin sensitive and resistant *Acinetobacter baumanii*, *E. coli*, and *K. pneumoniae*
**6.**	Artemisin	Plant (*Artemisia annua*)	*M. tuberculosis*	Inhibition of dormancy of *M. tuberculosis* [129]	Effective against malaria [130]
**7.**	Quercetin	Plant (such as red onion)	*E. coli*; *Vibrio parahaemolyticus; Listeria monocytogenes*	Biofilm inhibition [131]	Inhibitor of ROS production [132]
**8.**	*N*-(Heptylsulfanylacetyl)-l-homoserine lactone	Plant (garlic extract)	*P. aeruginosa*	AHL antagonist [132]	Decreased elaboration of virulence factors and reduced production of quorum sensing signals [133]
**9.**	Nitric oxide (NO)	Human, chemical source: Sodium nitroprusside (SNP) S-Nitroso-L-glutathione (GSNO) S-nitroso-N-acetylpenicillamine (SNAP)	*P. aeruginosa* and other pathogens	Dispersal of cells in the biofilm [134]	Vasodilation [135] Signaling molecule (reduction of cGMP) [134]
**10.**	Human Atrial Natriuretic Peptide (hANP), human Brain Natriuretic Peptide (hBNP), human C-type Natriuretic Peptide (hCNP)	Human	*P. aeruginosa*; *S. aureus*; *Cutibacterium. acnes*	Biofilm inhibition; biofilm dispersion; *S. aureus* biofilm inhibition and *Staphylococcus epidermidis* biofilm stimulation at 37 °C [136]	Opposite effect on S. *aureus* biofilm formation at 33 °C; Regulates blood pressure [137]
**11.**	Furanones Brominated furanones	Marine seaweed (*Delisea pulchra*)	*P. aeruginosa* *Salmonella enterica*	Targets quorum sensing systems; Inhibits certain virulence factors [138] Inhibition of quorum sensing [128]	Derivatives used in flavor and perfume industry [138] Cytotoxic
**12.**	*Cis*-2-decenoic acid *Trans*-2-decenoic acid *Cis*-11-methyl-2-dodecenoic acid	*P. aeruginosa*	*P. aeruginosa*	biofilm dispersal [139]	Autoinducer
			*Agrobacterium tumefaciens*	[140]	
			*S. aureus*	[141]	
			*C. albicans*	[142]	
**13.**	LL-37	Human	*P. aeruginosa* *E. coli*	Prevents biofilm formation; inhibits curli biosynthesis of *E. coli* [122,123]	Immunostimmulatory effects
**14.**	Alkaloids	*Aspergillus restrictus*	*C. albicans*	Inhibits growth of hyphae and production of extracellular polymeric substances [143]	Anticancer, anti-inflammatory, and analgesic properties
**15.**	Indolocidin	Isolated from bovine neutrophil cytoplasmic granules	*S. maltophilia, P. aeruginosa*	Inhibits biofilm formation [144]	-
**synthetic compounds**					
**16.**	1037	Antimicrobial peptide LL-37	*aeruginosa,* *L.monocytogenes* *Escherichia coli*	Biofilm inhibition/eradication [145] Antibiofilm properties [123]	Stimulation of twitching motility [146] Contributes to innate immunity [122]
**17.**	1018	Antimicrobial peptide Bactenecin	*P aeruginosa, E. coli, A. baumannii, Klebsiella pneumoniae, S. enterica* and MRSA	Biofilm inhibition/eradication [145]	Antibacterial properties (MIC > 256 μg/mL)
**18.**	AS10	Cathelicidin-Related Anti-Microbial Peptide CRAMP: LL37 analog	*C. albicans*	Biofilm inhibition [145]	-
**19.**	BMAP27-melittin	Peptide Melittin	*S. aureus, P.aeruginosa*	Biofilm inhibition/eradication [145]	ATP synthesis [146]
**20.**	NRC-16	Antimicrobial peptide Pleurocidin	*P. aeruginosa*	Biofilm inhibition [145]	Antibacterial properties MIC > 256 μg/mL and lowers hemolysis [147]
**21.**	Battacin	Lipopeptides	*P. aeruginosa, P. syringae, S. aureus*	Biofilm inhibition/eradication [145]	Antibacterial activity by depolarization of cell membrane [148]
**22.**	Complestatin (structural analog of vancomycin)	*Streptomyces* spp	*P. aeruginosa*	Inhibition of biofilm formation; upregulation of NO production; and reduction of cyclic di-GMP [149]	Antibacterial activity (analog of vancomycin) [149]
**23.**	Hydrazonodiaminopyrazole H6-335	3-Fluoroaniline (Precursor)	*P. aeruginosa*	Depletes cyclic di-GMP/disperses biofilm [150]	-
**24.**	Temporin PTa and Hp-MAP1 and Hp-MAP2	Animal (*Hylarana picturata*)	*K. pneumoniae, A. baumanii, S. aereus, E. coli*	Antibiofilm activity of analogs Hp-MAP1 and Hp-MAP2 [151]	Antimicrobial activity
**25.**	Clarithromycin	Macrolide erythromycin	*P. aeruginosa* *Other bacteria*	Prevents biofilm formation [81]	Antibacterial activity, immunomodulatory activity
**26.**	(Z)-4-(2-(3-Fluorophenyl)hydrazine ylidene)-5-imino-4,5-dihydro-1H-pyrazol-3-amine	Hydrazono-diaminopyrazole derivative	*P. aeruginosa*	Leads to biofilm dispersal by stimulation of a cyclic di-GMP phosphodiesterase [150]	-
**27.**	Benzophenone semicarbazone	Benzophenone semicarbazone derivative	*Candida parapsilosis*	Prevents biofilm formation (Nadeem, Shafeeq et al., manuscript in preparation)	-

## Data Availability

Not applicable.
